# Multiple pulmonary nodules with high metabolic activity: Potential benefit of multiple nodule biopsies by video-assisted thoracic surgery: A case report

**DOI:** 10.3892/etm.2013.1180

**Published:** 2013-06-25

**Authors:** JINLIN WANG, SHIYUE LI, JUN LIU, YINGYING GU, PING CHEN

**Affiliations:** 1 Departments of Respiratory Medicine; 2 Cardiothoracic Surgery; 3 Pathology and; 4 Radiology, The First Affiliated Hospital of Guangzhou Medical University, Guangzhou Medical University, Guangzhou, Guangdong 510120;; 5 The State Key Laboratory of Respiratory Disease, Guangzhou Institute of Respiratory Disease, Guangzhou, Guangdong 510120, P.R. China

**Keywords:** pulmonary nodules, video-assisted thoracic surgery, lymphoepithelioma-like carcinoma, tuberculosis

## Abstract

The aim of this study was to assess complex cases of multiple pulmonary nodules (PNs) with high metabolic activity that may have benefited from being managed in a manner outside the formal guidelines. The study describes the case of a patient with multiple highly metabolically-active PNs, where an original diagnosis of lung cancer metastasis was proposed. Following a failed transbronchial lung biopsy (TBLB), multiple nodule biopsies by video-assisted thoracic surgery (VATS) were performed, and a diagnosis of lymphoepithelioma-like carcinoma (LELC; stage IA) and tuberculosis was reached. This case report demonstrated that multiple nodule biopsies by VATS were effective and were able to improve the prognosis without delay.

## Introduction

A pulmonary nodule (PN) is defined as a spherical, radiographic opacity <3 cm in diameter that is entirely surrounded by lung tissue (1,2). ^18^F-fluorodeoxyglucose positron emission tomography/computed tomography (^18^F-FDG-PET/CT) has been widely used in the differential diagnosis of multiple PNs, since it is able to effectively detect any PNs, as well as nodules elsewhere in the body, in addition to monitoring the metabolic status of the nodules (3,4). PNs with a high metabolic activity are often considered to be metastatic (5). According to the clinical practice guidelines of the American College of Chest Physicians (ACCP) (6), the current therapeutic strategies for patients with multiple highly metabolically active PNs include radiographical follow-up, tissue sampling or surgical resection. Clinicians are required to discuss the risks and benefits of alternative management strategies and elicit patient preferences; however, there is no general consensus with regard to the optimal management of cases, which results in certain challenges.

The current study describes the case of a patient with multiple PNs with high metabolic activity, where an initial diagnosis of lung cancer metastasis was proposed. However, it was not possible to obtain sufficient clarification of the diagnosis through conventional methods, including transbronchial lung biopsy (TBLB), and therefore multiple nodule biopsies were performed by video-assisted thoracic surgery (VATS). This resulted in an unexpected diagnosis, which entailed a better prognosis and a change in the therapeutic strategy. The aim of this study was to assess the role of multiple nodule biopsies by VATS in the diagnosis of multiple highly metabolically active PNs in the context of a case observed in The First Affiliated Hospital of Guangzhou Medical University (Guangzhou, China). Written informed consent was obtained from the patient for the publication of this case report and any accompanying images.

## Case report

**Figure 1.**
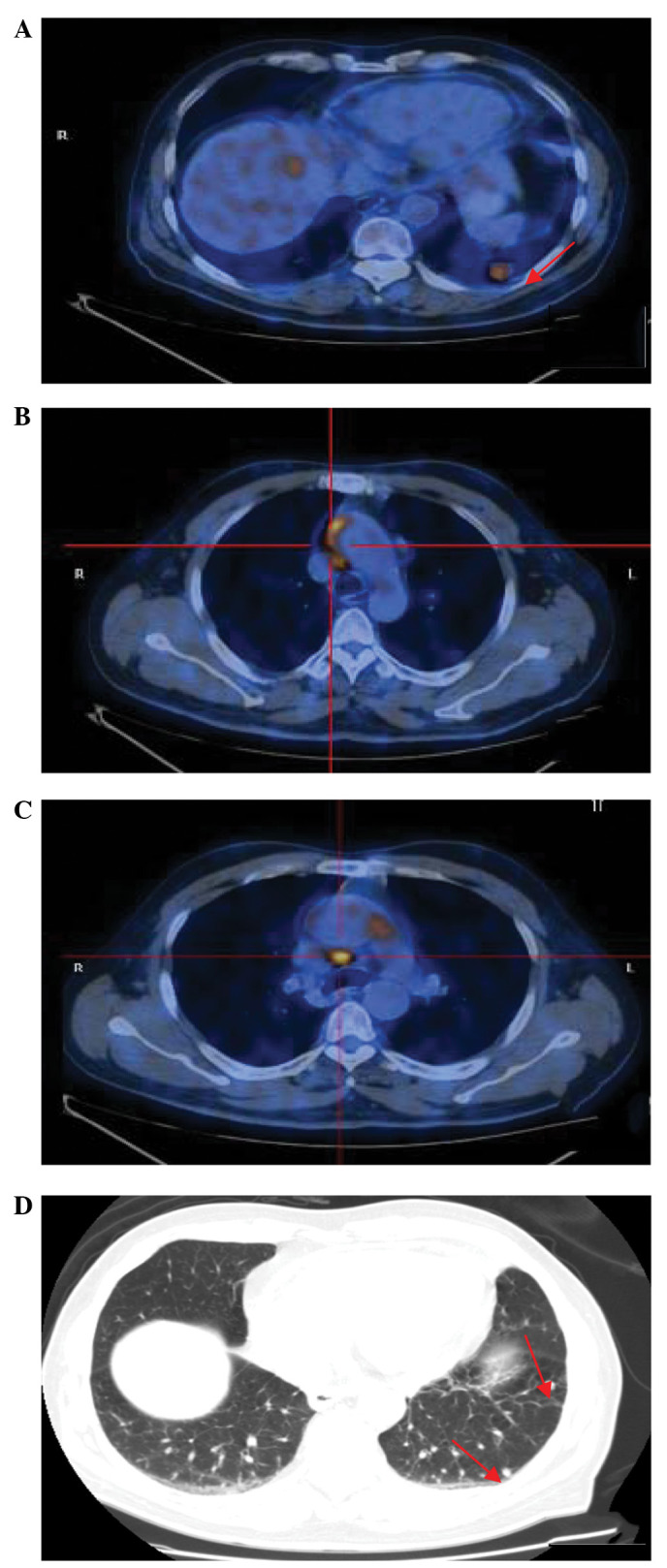
Nodules revealed by ^18^F-fluorodeoxyglucose positron emission tomography/computed tomography (^18^F-FDG-PET/CT) (August 3, 2011). (A) The largest nodule is visible at the basal segment of the left lower lobe [maximum standardized uptake value (SUV_max_), 5.8]. (B) Nodules observed in the ascending aorta/aortic arch wall (SUV_max_, 6.4). (C) Nodule observed in the pericardial wall (SUV_max_, 8.3). (D) Nodules observed in the left lower lobe (^18^F-FDG uptake was normal).

A 70-year-old male was admitted to The First Affiliated Hospital of Guangzhou Medical University due to a cough with sputum that had been present for half a month. The patient had a 40-year smoking history. A physical chest examination did not reveal any significant signs of abnormalities and the results of laboratory tests were not indicative of a specific diagnosis. A computed tomography (CT) scan of the chest revealed multiple PNs, with the largest nodule measuring 1.9 cm in its maximum dimension. This nodule was located at the basal segment of left lower lobe. When no improvement was observed following a one-week course of antibiotics, an ^18^F-FDG-PET/CT scan was performed, which revealed an increased uptake in the largest pulmonary nodule, with a maximum standardized uptake value (SUV_max_) of 5.8 (Fig. 1A). The two foci were located in the ascending aorta/aortic arch wall and pericardial wall with SUV_max_ values of 6.4 and 8.3, respectively (Fig. 1B and C). In addition, mediastinal lymph nodes with an SUV_max_ of 2.5 and other PNs with normal uptakes were observed (Fig. 1D). These observations resulted in a diagnosis of lung cancer metastasis being proposed. Following this, a bronchoscopy and a TBLB, guided by X-ray, were performed; however, no positive result was indicated.

**Figure 2.**
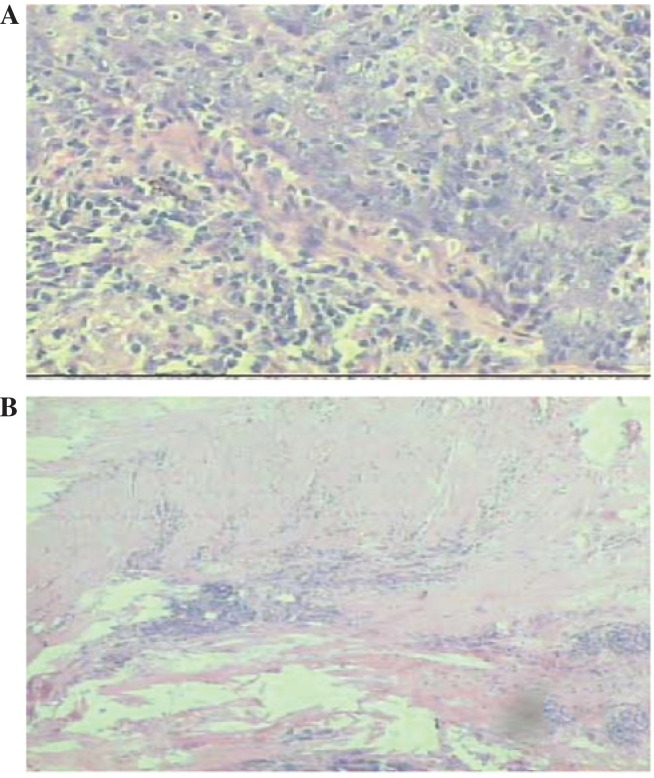
Pathological section of the nodules. (A) Tumor cells are arranged in nests and are rich in cytoplasm, with nucleoli evident in the largest nodule. Lymphocytes are present in the background [hematoxylin and eosin (H&E) staining; magnification, ×400). (B) Tuberculoid nodule, with hyaline degeneration and surrounding lymphoid tissue hyperplasia (H&E staining; magnification, ×100).

To obtain a definitive diagnosis and an appropriate treatment, a VATS lung biopsy was performed, following the provision of signed informed consent from the patient. Three nodules, including the largest nodule, a nodule at the lingular segment of the left upper lobe and a nodule at the pericardial wall, were completely enucleated during one surgical session under the same anesthesia The histological results of the frozen sections obtained from the nodules intraoperatively revealed that the largest nodule was a lung carcinoma, while the remaining nodules were indicative of tuberculosis. The standard treatment of lobectomy with systematic mediastinal, hilar and interlobar lymphadenectomies was completed by VATS. The pathological sections and immunohistochemistry confirmed a diagnosis of lymphoepithelioma-like carcinoma (LELC) (stage pT_1_N_0_M_0_ IA) in the largest nodule (Fig. 2A), while the two remaining small PNs and the pericardial nodule were confirmed as tuberculoid, with observations of hyaline degeneration and hyperplasia of the surrounding lymphoid tissue (Fig. 2B), indicating obsolete or active tuberculosis.

**Figure 3.**
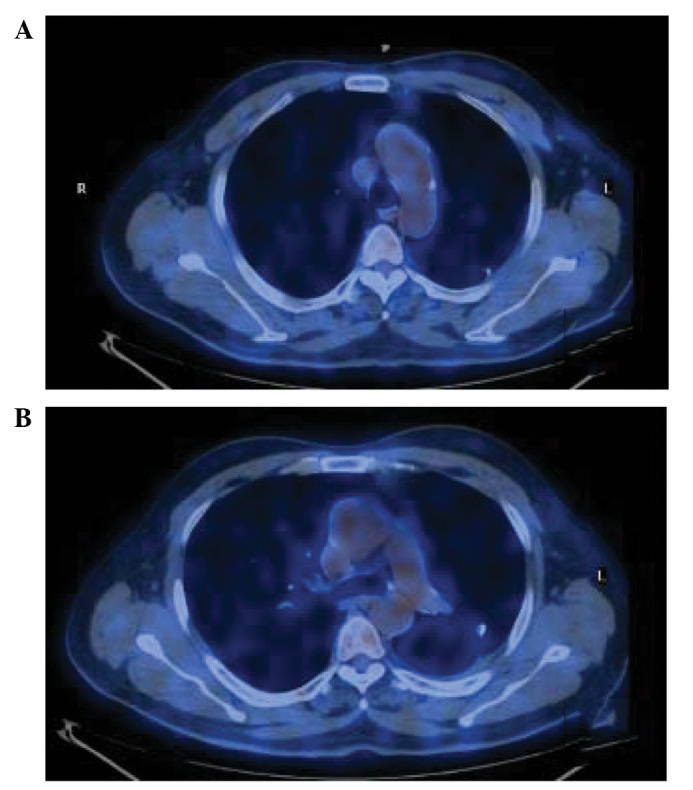
Follow-up of ^18^F-fluorodeoxyglucose positron emission tomography/computed tomography (^18^F-FDG-PET/CT) (December 3, 2011). (A) Nodules in the ascending aorta/aortic arch wall have disappeared (normal ^18^F-FDG uptake), (B) as has the nodule in the pericardial wall (normal ^18^F-FDG uptake).

Two weeks subsequently, the patient was treated with anti-tuberculous drugs. In the six months of follow-up, the patient did not present with any symptoms and ^18^F-FDG-PET/CT revealed that the remaining PNs were stable, with no change in size, the nodules of the ascending aorta/aortic arch and pericardial wall had disappeared and the ^18^F-FDG uptakes were normal (Fig. 3).

## Discussion

Multiple highly metabolically active PNs (in addition to nodules elsewhere in the body) are common in clinical practice, with the PNs frequently resulting in a differential diagnosis of lung cancer metastasis (7–9). The efficacy of ^18^F-FDG-PET/CT in the differentiation of benign and malignant PNs >1 cm has been investigated in a number studies (3,4); however, there is a high occurrence of false negative results for nodules <1 cm with regard to highly differentiated adenocarcinoma and slowly progressive malignant tumors (10,11). Furthermore, ^18^F-FDG-PET/CT is not able to identify lung cancer in combination with other metabolic diseases (12). Therefore, the presence of multiple PNs with high metabolic activity is always associated with diagnostic and therapeutic challenges. In the present case, taking into consideration factors such as age, smoking history, symptoms, treatment and the result of the PET/CT scan, lung cancer metastasis was the primary conclusion. Therefore, there is a requirement for the employment of precise methods to ensure the early discovery of malignant nodules, in order to improve the prognosis.

The main aims of nodule treatment include the identification of malignant nodules at the earliest opportunity and the avoidance of the surgical treatment of benign nodules (5,13). TBLB is a moderately invasive technique; however, the sensitivity, guided by radial probe endobronchial ultrasonography (14) or electromagnetic navigation bronchoscopy (15), has potential for improvement. For peripheral nodules, the sensitivity of transthoracic needle aspiration (TTNA) is higher than that of TBLB, and has been observed to vary from 70 to 100% (16,17). However, challenges become apparent when the results are negative; furthermore, it is easy to ignore the coexistence of other diseases. This may lead to misdiagnosis and diagnostic errors when one nodule has a positive pathology (15). In the present study, following the failure of the TBLB to provide a positive result, it was relatively difficult to perform a biopsy by TTNA and somewhat easier to consider a diagnosis of lung cancer metastasis and then delay the treatment if a positive pathological result was obtained from the TBLB. Thus, TBLB and TTNA exhibit numerous limitations with regard to the diagnosis of multiple highly metabolically active PNs.

A VATS lung biopsy possesses fundamental advantages for the diagnosis of multiple PNs with high metabolic activity. It reduces the surgical trauma, the duration of the hospital stay, the postoperative pain and the time required for the complete recovery of the patients’ normal activity. Furthermore, there have been no intra- or postoperative mortalities or significant intraoperative complications observed with the procedure (18). The biopsy is performed under general anesthesia using lung ventilation via a double-lumen endotracheal tube. Recently, it has been demonstrated that the VATS may be performed under anesthesia with nortraceal intubation (19). It provides excellent visualization of the entire lung surface, chest wall and mediastinum; moreover, it enables multiple nodule biopsies to be performed during one surgical session. The samples obtained provide the potential for the acquisition of a fast, certain and definitive diagnosis by intraoperative frozen section histology. As the procedure has been used more extensively, the range of detectable nodule diameters has expanded, reportedly varying between 3 and 30 mm (18). When the detection of subpleural nodules is difficult due to the nodules being too small or too far from the pleural surface, the placement of a preoperative CT-guided marking using a hookwire with a string may be done promptly, to enable the VATS lung biopsy (20). However, for patients with lung cancer metastasis, the surgery is only a means for providing a diagnosis; therefore, a conservative treatment option is always likely to be selected by the patients. In the present study, taking into consideration the age, trauma, economy and the initial diagnosis of lung cancer metastasis, the patient was not willing for the VATS to be conducted. After the patient was persuaded to reconsider and agree to undergo the VATS, a VATS lung biopsy was performed for the different nodules and a diagnosis of LELC and tuberculosis was reached. The standard method of treatment, i.e. lobectomy with systematic mediastinal, hilar and interlober lymphadenectomies, was then implemented, which avoided delay and improved the prognosis of the patient.

In conclusion, in the present study the VATS lung biopsy was demonstrated to be a safe and effective procedure that enabled an accurate diagnosis through multiple nodule biopsies in a minimally invasive manner. The procedure was particularly beneficial in this case, since it avoided excessive instrumental examinations, misdiagnoses and inappropriate treatments.
